# Constitutive Model and Microstructure Evolution of Ti65 Titanium Alloy

**DOI:** 10.3390/ma17102409

**Published:** 2024-05-17

**Authors:** Tao Sun, Lili Sun, Haihao Teng, Wenhao Liu, Ruiqi Wang, Xuanjie Zhao, Jie Zhou

**Affiliations:** 1 College of Materials Science and Engineering, Chongqing University, Chongqing 400044, China; 20210901056@cqu.edu.cn (T.S.); 202309021114t@stu.cqu.edu.cn (L.S.); 20210901006@stu.cqu.edu.cn (H.T.); wangruiqi15@foxmail.com (R.W.); 20230901093g@stu.cqu.edu.cn (X.Z.); 2 College of Physics, Sichuan University, Chengdu 610064, China; sculiuwenhao@163.com; 3 Deyang Wanhang Die Forging Co., Ltd., China National Erzhong Group Co., Deyang 618000, China

**Keywords:** constitutive equation, Ti65 alloy, machine-learning algorithm, hot deformation

## Abstract

The hot deformation behavior and mechanism of Ti65 alloy with a bimodal microstructure were investigated by isothermal compression experiments conducted on the Thermecmastor-Z simulator equipment at temperatures ranging from 950 to 1110 °C and strain rates ranging from 0.01 to 10.0 s^−1^. The Arrhenius constitutive model, based on strain compensation, and Grey Wolf optimization-neural network with back propagation model (GWO–BP), were both established. The differences between the experimental and predicted value of flow stress were compared and analyzed using the two models. The results show that the prediction accuracy of GWO–BP in the two-phase region is higher than that of Arrhenius model. In the single-phase region, both methods demonstrated high prediction accuracy. Compared to the single-phase region, the flow stress of Ti65 alloy shows a higher degree of softening in the two-phase region. During deformation in the two-phase region, the initial lamellar α phase transformed from a kinked and elongated morphology to a globularized topography as the strain rate decreased. Boundary-splitting was the primary mechanism leading to the spheroidization process. The degree of recrystallization increased with the increase in strain rate during the deformation in the single-phase region, while dynamic recovery and strain-induced grain boundary migration were the main deformation mechanisms at a lower strain rate. Discontinuous dynamic recrystallization may be the dominant recrystallization mechanism under a high strain rate of 10 s^−1^.

## 1. Introduction

Titanium alloys are commonly produced by incorporating diverse elements, like aluminum, vanadium, and molybdenum, into the liquid titanium phase. These titanium alloys are well known for their remarkable specific strength and their exceptional heat and chemical resistance, making them highly coveted for a diverse array of industrial applications [1,2]. Among various titanium alloy types, near-α titanium alloys have garnered considerable attention owing to their combination of high thermal stability, fatigue, and creep properties at high ambient temperatures [3]. The microstructure of titanium alloy determines its mechanical properties, while it can be affected by the deformation parameters and heat treatment process, so it is very important to understand the relationship between microstructure, processing parameters and mechanical properties. To meet the requirements on the performance of near-α titanium alloys under a stringent service environment, thermomechanical processing (TMP) is usually employed to obtain optimized microstructures for the desired final service properties. Forging, equivalent to hot compression deformation, is a primary TMP method for manufacturing large-scale components due to its dimensional stability and high production efficiency. The deformation mechanisms exhibited by titanium alloys vary distinctly based on specific deformation parameters. Dynamic recovery, dynamic recrystallization and dynamic spheroidization are the main thermal deformation mechanisms of titanium alloys [4]. Under certain deformation conditions, flow localization, micro-shear bands and dynamic spheroidization can also be observed [5]. This certainly complicates the operation of selecting the appropriate deformation conditions. Therefore, a systematic analysis of the evolution of microstructure during deformation of titanium alloys is the key to obtaining products with qualified mechanical properties.

Accurate numerical simulation of plastic deformation processes requires a deep understanding of the complex relationship between the rheological stress values and deformation variables of the material, as well as an in-depth knowledge of the deformation mechanism and microstructure. Currently, the establishment of a constitutive model is a reliable approach for predicting the thermal deformation behavior. Recently, the development and utilization of constitutive models have been extensively employed across a wide range of materials [6,7,8,9]. The constitutive model, microstructure and thermal deformation behavior and mechanism of titanium alloy have been studied extensively. Based on a thermal compression experiment, the thermal deformation behavior and microstructure evolution of Ti6554 were systematically analyzed, the corresponding constitutive model and hot processing map were established, and the recrystallization mechanism under different deformation states was further analyzed in detail [10]. The microstructure and texture evolution during the thermal deformation of Ti5321 were studied in detail. It was found that the deformation mechanism in the single-phase region was continuous dynamic recrystallization, while dynamic spheroidization of the α-phase occurred mainly in the two-phase region. In addition, <100> + <111>//CD were always the two main fiber textures during the hot compression of this alloy [11]. Zhao et al. [12] employed various heat treatment parameters to regulate the grain size and structure, and explored the correlation between silicide precipitation and properties in Ti65 alloy. The constitutive models of Ti-5Al-3Mo-1.5V under thermal deformation conditions in different phase regions were established, and the appropriate processing parameters were identified according to the calculated hot processing map. The relationship between the grain size and strain rate was further discussed and analyzed [13]. These comprehensive investigations demonstrate that the integration of a constitutive model with microstructure analysis offers valuable insights for determining optimal deformation process parameters and accurately predicting hot deformation behavior. At present, for wrought Ti65 high temperature titanium alloys, especially those with an initial bimodal microstructure, there is no research on the constitutive model, microstructure evolution, and the relationship between the thermal deformation mechanism and deformation parameters. The establishment of a constitutive model for Ti65 alloy is helpful for accurate simulation of its hot working process. Further analysis of its microstructure evolution and deformation mechanism, and exploration of the relationship between deformation parameters and microstructure evolution, are necessary to expand the application range of the alloy in industry.

The Ti65 alloy with bimodal microstructure was investigated by isothermal compression experiments at temperatures ranging from 950 (two-phase region) to 1110 °C (single-phase region) and strain rates ranging from 0.01 to 10.0 s^−1^. The purpose of this study was to establish a constitutive model to accurately predict the flow stress, analyze the microstructure evolution under different deformation parameters, and explore the main softening mechanism in different phase zones of Ti65 alloy. In this study, the microstructure analysis mainly relies on optical metallography, scanning electron microscopy and electron backscatter diffraction analysis. The relevant results of this research have important theoretical and practical significance for numerical simulation analysis, precise control of the microstructure of Ti65 alloy and the ultimate guarantee of qualified mechanical properties and can expand the industrial application of Ti65 high temperature titanium alloy to a certain extent.

## 2. Materials and Experimental Methods

### 2.1. Experimental Material

**Figure 1:**
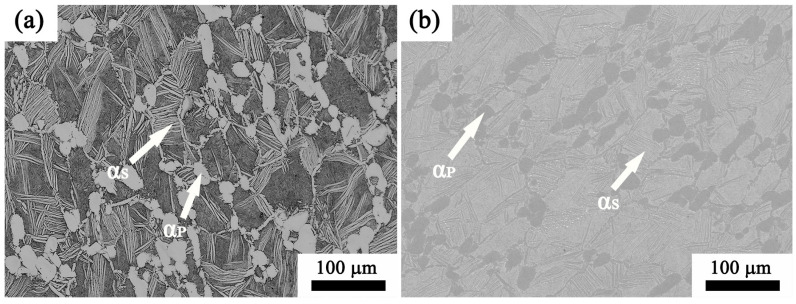
Initial microstructure of Ti65 alloy, including primary α phase (α_p_) and lamellar α phase (α_s_): (**a**) optical metallography image, (**b**) scanning electron microscopy image.

**Figure 2:**
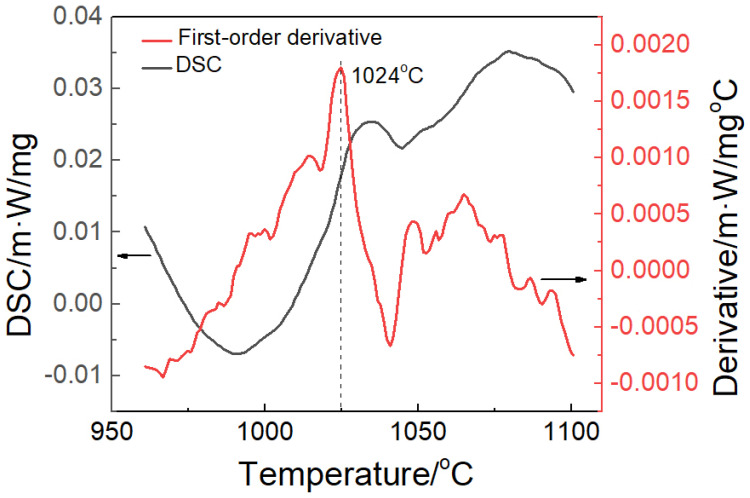
DSC and first-order derivative curves of Ti65 titanium alloy.

The as-received Ti65 high-temperature titanium alloy is forged billet supplied by Deyang Wanhang Die Forging Co., Ltd. in Deyang, China, and has undergone forging with a sequence of procedures for the single-phase and two-phase regions. The Ti65 alloy has the following measured composition: 5.92% Al, 3.94% Sn, 3.45% Zr, 0.86% Ta, 0.66% W, 0.48% Si, 0.32% Nb, 0.31% Mo, 0.05% C, and the remaining balance is Ti (wt. %), as listed in Table 1. Figure 1a,b illustrates the initial microstructure of the as-received Ti65 alloy. It can be seen from Figure 1 that the initial Ti65 is a bimodal structure, including an approximately equiaxial primary α phase (α_p_) and lamellar α phase arranged in colonies (α_s_), which can be indicated by white arrows. According to the DSC analysis results in Figure 2, the phase transition point of Ti65 alloy is 1024 °C.

### 2.2. Method

**Figure 3:**
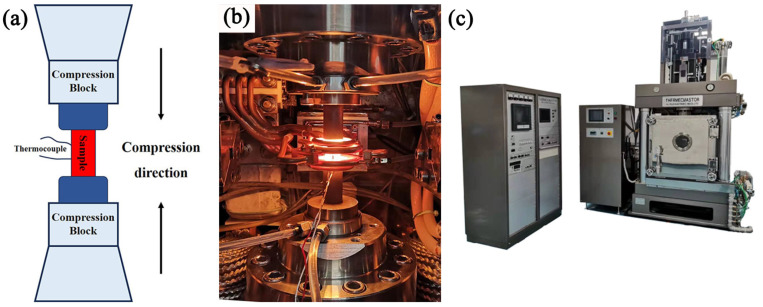
Isothermal compression experiment: (**a**) schematic diagram of thermal compression experiment, (**b**) on-site production image of the compression experiment, (**c**) Thermecmastor-Z compressor equipment.

**Figure 4:**
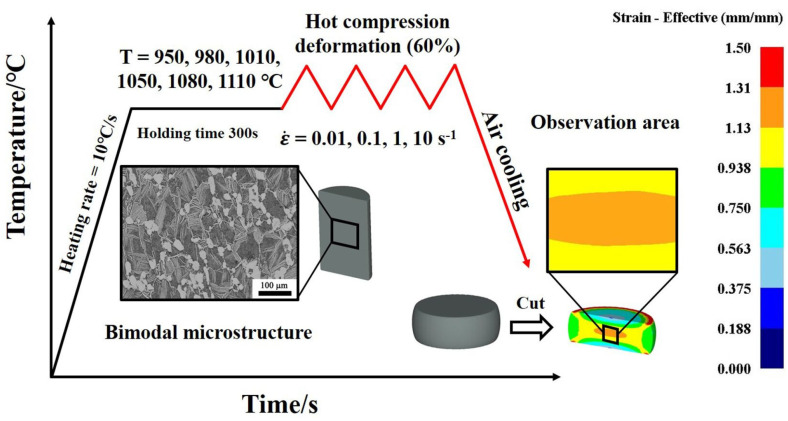
Experimental schematic diagram and observation area of the sample during the hot compression of Ti65 alloy.

Compression tests were implemented on a Thermecmastor-Z thermal simulator to study the hot working characteristics, and microstructural evolution, and establish the constitutive model of the Ti65 alloy. Figure 3 shows the schematic diagram of the thermal compression process and a picture of the experimental equipment used in the research. Initially, the Ti65 billet, which had been forged, was processed using wire-cutting techniques to create cylindrical specimens measuring Φ8 × 12 mm. The axial direction of the cylindrical specimen is consistent with the forging direction of the initial billet. A concentric groove with a depth of 0.3 mm is fabricated on each of the upper and lower sides of the compression samples. The grooves serve to retain the lubricant during the deformation of the specimen, mainly to minimize the friction between the specimen and the anvil. To prevent the oxidation of the sample surface, it is essential to introduce argon gas into the equipment as a protective environment prior to heating the sample. The real strain reached 0.9, corresponding to a deformation amount of 60%. The heating rate of the hot compression sample was set at 10 °C/s. Subsequently, they were maintained at those temperatures for 300 s to establish thermal equilibrium prior to the commencement of the test. The specimens after deformation were immediately cooled with air to maintain the microstructure and the center area with the largest amount of deformation was used for microstructural observation. The experimental schematic diagram is shown in Figure 4.

### 2.3. Microstructure Analysis

The samples for metallographic and scanning electron microscope analysis were first grinded, then mechanically polished, and finally etched with a mixture of HF + HNO_3_ + H_2_O (volume ratio is 1:3:96) for 10 s. The polished surface was promptly rinsed with water and dried after etching. In preparation for the EBSD test, the surfaces underwent a two-step polishing process. First, they were mechanically polished. Then, they were electrolytically polished using a mixture etching solution of perchloric acid, n-butanol and carbinol (volume ratio is 5:35:60) under 30 V, at −35 °C. The EBSD experiments were performed on a 7800F scanning electron microscope equipped with a high-resolution Oxford Instrument probe for receiving diffraction pattern signals. The scanning step size during the data acquisition is set to 0.25 µm. Aztec Crystal (Version 2.12) was used as the offline data processing software (Oxford Instruments, Abingdon, Oxfordshire, UK).

## 3. Results and Discussion

### 3.1. Ture Strain-Stress Analysis

**Figure 5:**
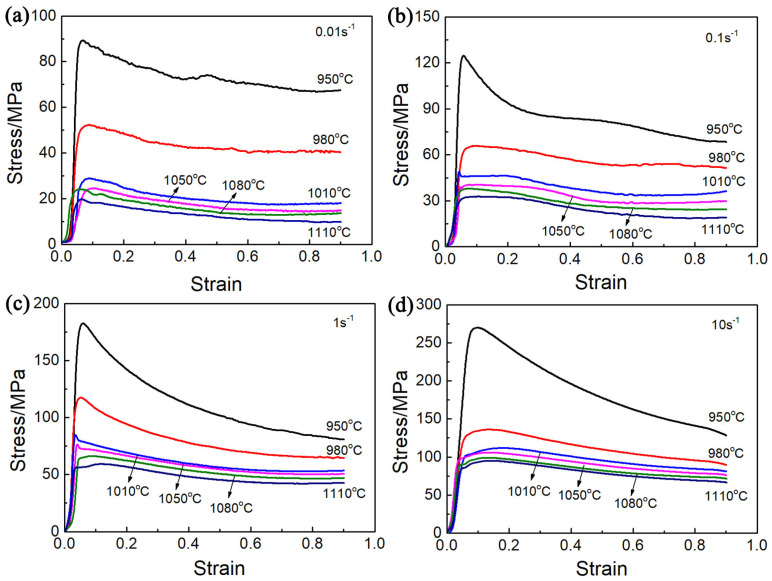
Ture stress–strain curves under different thermal compression conditions: (**a**) 0.01 s^−1^, (**b**) 0.1 s^−1^, (**c**) 1 s^−1^, and (**d**) 10 s^−1^.

**Figure 6:**
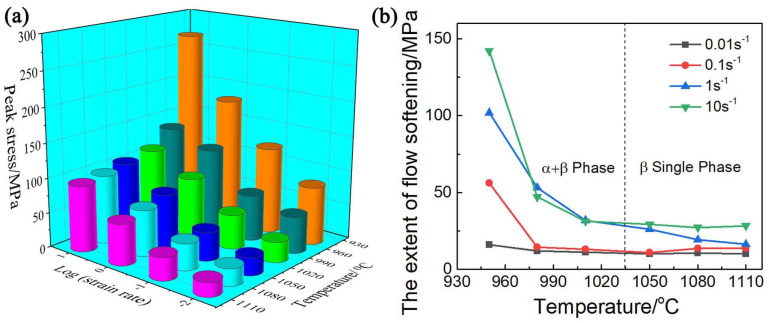
(**a**) Relationship between the peak stress and thermal deformation parameters, (**b**) relationship between the extent of flow softening and deformation parameters of Ti65 alloy.

The relationship curves between true stress and strain obtained for Ti65 alloys for various deformation states, are shown in Figure 5. Due to the work-hardening effect, the flow stress rises rapidly in the early stages of compression until it reaches a maximum value in the small strain state [14,15]. The results show that the initial strength increase in the stress–strain curve is primarily attributed to the generation, migration, and proliferation of dislocations [10,16]. At the same time, dynamic recovery, dynamic recrystallization and spheroidization of α phase are difficult to occur at the initial stage of deformation [17,18]. The maximum values of stress also vary depending on the deformation parameters, as illustrated in Figure 6a. With the increase in temperature and decrease in strain rate, the peak stress shows a decreasing trend. The reason for this phenomenon is that the rate of dislocation propagation is significantly higher at high strain rates, which leads to greater work-hardening effects and ultimately to higher rheological stress [19]. On the other hand, with the increase in temperature, more α phase transforms into β phase. β phase has more slip systems than α phase, and exhibits higher deformability, resulting in obvious softening effect. Figure 6b illustrates the flow softening effect ($∆\sigma =\sigma_{P}-\sigma_{0.9}$) [20] for various deformation conditions. The softening degree of Ti65 alloy decreases with increasing temperature and decreasing strain rate, and the softening tendency of the alloy in the two-phase region is more obvious than that in the single-phase region, which aligns with the findings of other researchers [21,22]. The fluctuation in the degree of flow softening, as temperature rises and strain rate decreases, is ascribed to the evolution of microstructure, deformation mechanism and deformation heating, such as variations in grain morphology and size, dislocation fraction, and phase composition [23,24,25]. Discontinuous yielding phenomenon (DYP) was observed at strain rates of 0.1 and 1 s^−1^ and temperatures of 1010 °C and 1050 °C. At the beginning of the deformation, a large accumulation of dislocations occurs along the grain boundaries, leading to a sudden increase in the value of a flow stress. Once the dislocation density exceeds a specific threshold, pinned dislocations cross the boundary, causing the elimination of dislocations and subsequent decrease in flow stress [26].

### 3.2. Arrhenius Constitutive Model Considering Strain Compensation

The flow stress is mainly related to temperature, deformation rate, deformation amount and other parameters. Scientists have been working to develop intrinsic models that accurately describe the correlation between the flow stress and processing variables during deformation [27]. The Arrhenius constitutive model, expressed as a hyperbolic sine function, is considered an effective method for establishing the relationship between the flow stress and deformation parameters [28], displayed as follows:

(1) $$\dot{\varepsilon }={A[\sin h\left(\alpha \sigma \right)]}^{n}\exp(-Q/\mathrm{RT}),$$

where the symbol $\dot{\varepsilon }$ represents the strain rate, and σ represents the flow stress. *A*, *α*, and *n* are material variables that do not depend on temperature.

Equation (1) has the following two expressions depending on the stress state:

(2) $$\dot{\varepsilon }=A_{1}\sigma^{n_{1}}\exp\left(-Q/\mathrm{RT}\right)\;(\alpha \sigma \;\leq \;0.8)$$

(3) $$\dot{\varepsilon }=A_{2}\exp(\beta \sigma )\exp(-Q/\mathrm{RT})\;(\alpha \sigma \;\geq \;1.2)$$

where $A_{1}=A\alpha^{n_{1}}$, $A_{2}=A/2^{n}$, $\beta =\alpha n_{1}$.

Equations (2) and (3) could be further formulated as:

(4) $$\ln\dot{\varepsilon }=\ln A_{1}-Q/\mathrm{RT}+n_{1}\ln\sigma$$

(5) $$\ln\dot{\varepsilon }=\ln A_{2}-Q/\mathrm{RT}+\beta \sigma$$

where n1=∂ln⁡ε˙∂ln⁡σ and β=∂ln⁡ε˙∂σ in Equations (4) and (5). To calculate all the material variables effectively, the maximum stress values in various phase fields were inserted into Equations (4) and (5).

**Figure 7:**
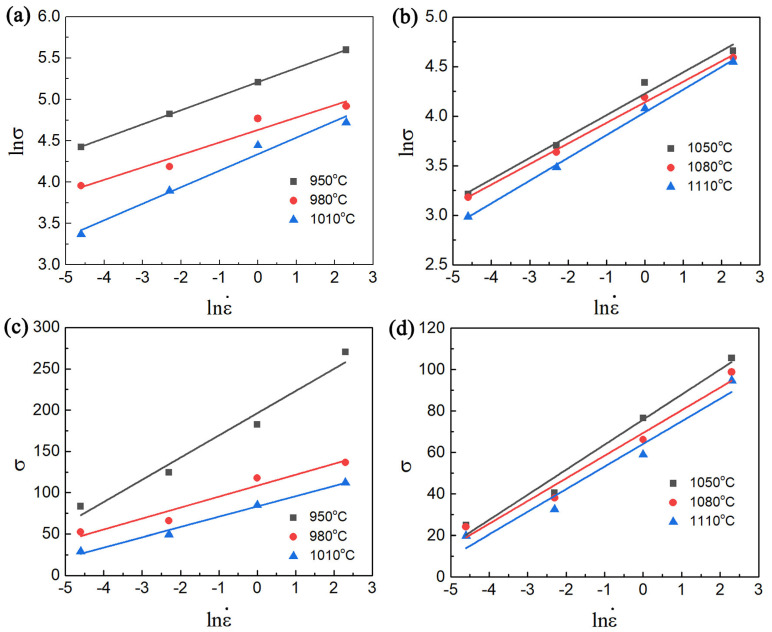
The correlations curves: (**a**) $\ln\sigma \;\mathrm{and}\;\ln\dot{\varepsilon }$ in the α + β phase field, (**b**) $\ln\sigma \;\mathrm{and}\;\ln\dot{\varepsilon }$ in the single β phase field, (**c**) $\sigma \;\mathrm{and}\ln\dot{\varepsilon }$ in the α + β phase field, (**d**) $\sigma \;\mathrm{and}\ln\dot{\varepsilon }$ in the single β phase field.

Based on the data presented in Figure 7, the values $n_{1}$ and β in various phase regions can be determined by fitting the average inclinations of the relationships between $\ln\sigma -\ln\dot{\varepsilon }$ and $\sigma -\ln\dot{\varepsilon }$. Subsequently, the parameter α can be ascertained using the equation α=βn1. The values of α for the present alloy are 0.0099 and 0.0193 in the α + β phase field and single β phase field, respectively.

In a certain temperature range, *Q* can be regarded as a temperature-independent constant. Through formula transformation and derivation, the *Q* value can be calculated using the following formula:

(6) $$Q=R[\partial ln\dot{\varepsilon }/\partial ln[sinh(\alpha \sigma )]{]}_{T}[\partial ln[sinh(\alpha \sigma )/\partial (1/T){]}_{\dot{\varepsilon }},$$

**Figure 8:**
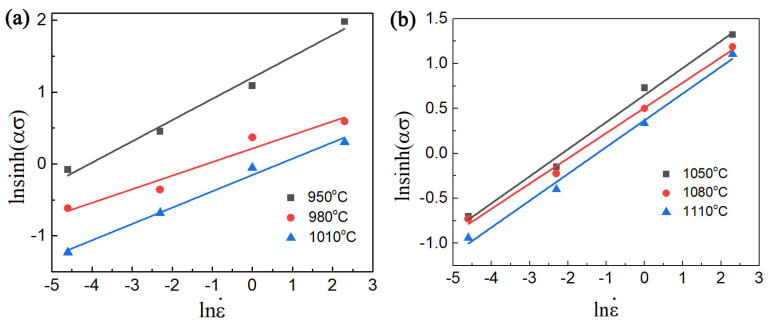
The correlation curves between $\ln[sinh(\alpha \sigma )]\;\mathrm{and}\;\ln\dot{\varepsilon }$ in different phase regions in (**a**,**b**).

**Figure 9:**
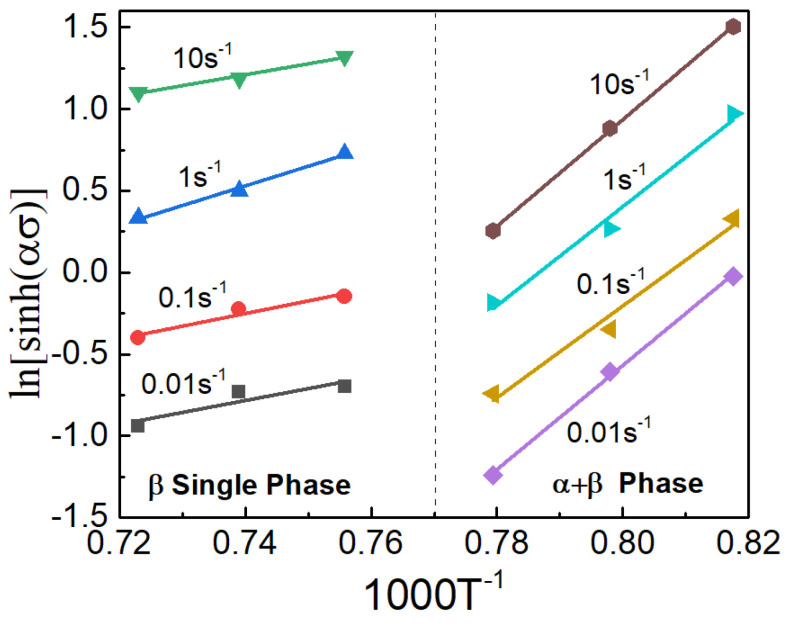
The correlation curves between $\ln\left[\sinh\left(\alpha \sigma \right)\right]\;\mathrm{and}\;{1000\;\times \;T}^{-1}$ in different phase regions.

And the calculation of the $\partial ln\dot{\varepsilon }/\partial ln[sinh(\alpha \sigma )$ can be performed by fitting the average inclinations of $\ln[sinh(\alpha \sigma )]-\ln\dot{\varepsilon }$, as seen in Figure 8a,b. Moreover, the computation of the second term is easily deduced from Figure 9.

The deformation activation energy *Q* was calculated to be 1172.167 and 238.728 kJ/mol in the two-phase field and single-phase field, respectively. Researchers [9,29,30] have suggested considering the effect of temperature on strain rate perturbation. The Z parameter can be described by the following formula:

(7) $$Z=A[sinh(\alpha \sigma ){]}^{n}=\dot{\varepsilon }exp\left(\frac{Q}{RT}\right)$$

**Figure 10:**
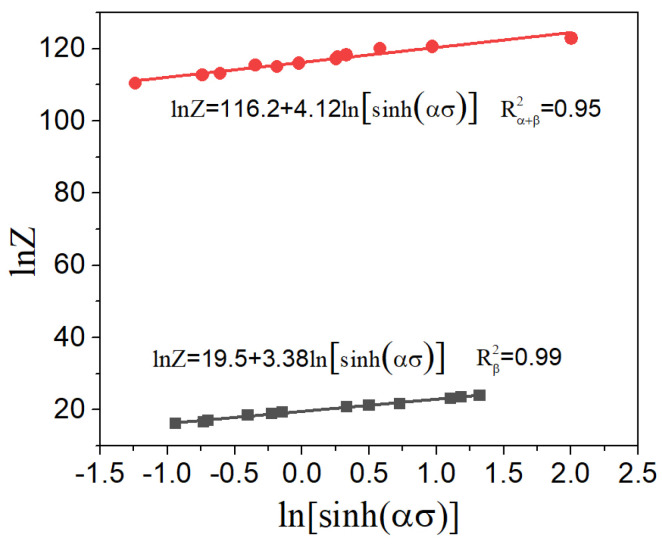
The correlation curves between ln⁡Z-$ln\left[sinh(\alpha \sigma )\right]$ in different phase regions.

Next, applying the natural logarithm function to Equation (8) can be carried out as follows:

(8) $$\ln Z=\ln A+n\ln\left[\sinh\left(\alpha \sigma \right)\right]$$

and the value of ln⁡A may be calculated by fitting the curves shown in Figure 10. The value of *n* can be obtained by the same method.

According to the above-mentioned analysis, the Arrhenius constitutive model of Ti65 alloy could be calculated by the following equations:

(9) $$\dot{\varepsilon }=1.32\times 10^{48}{\left[\begin{array}{c} \sinh\left(0.0099\sigma \right) \end{array}\right]}^{4.216}exp\left(-\frac{1172167}{RT}\right),$$

for the two-phase region, and

(10) $$\dot{\varepsilon }=2.95\times 10^{8}{\left[\begin{array}{c} \sinh\left(0.0193\sigma \right) \end{array}\right]}^{3.406}exp\left(-\frac{238728}{RT}\right),$$

for the single β phase region.

**Figure 11:**
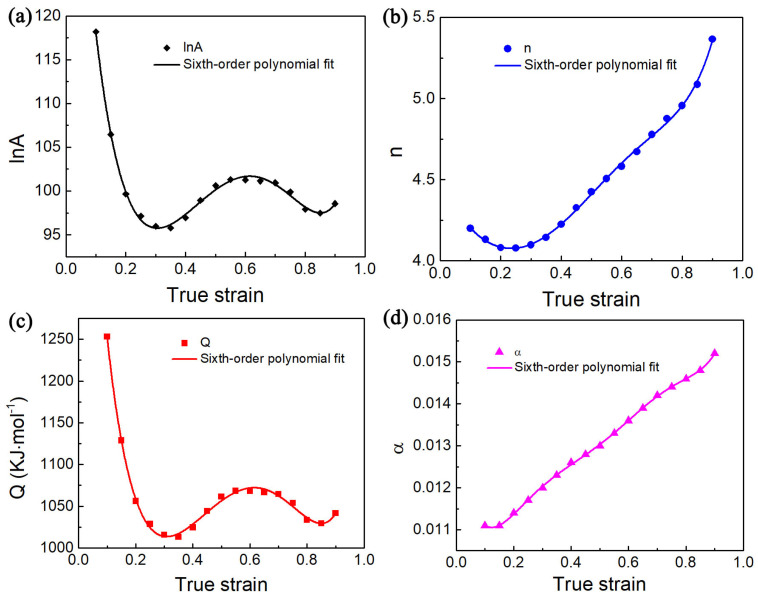
Correlation fitting curves between four material parameters ((**a**) ln *A*; (**b**) *n*; (**c**) *Q* and (**d**) *α*)) and strain value by employing the sixth-order polynomial fitting method in the two-phase region.

**Figure 12:**
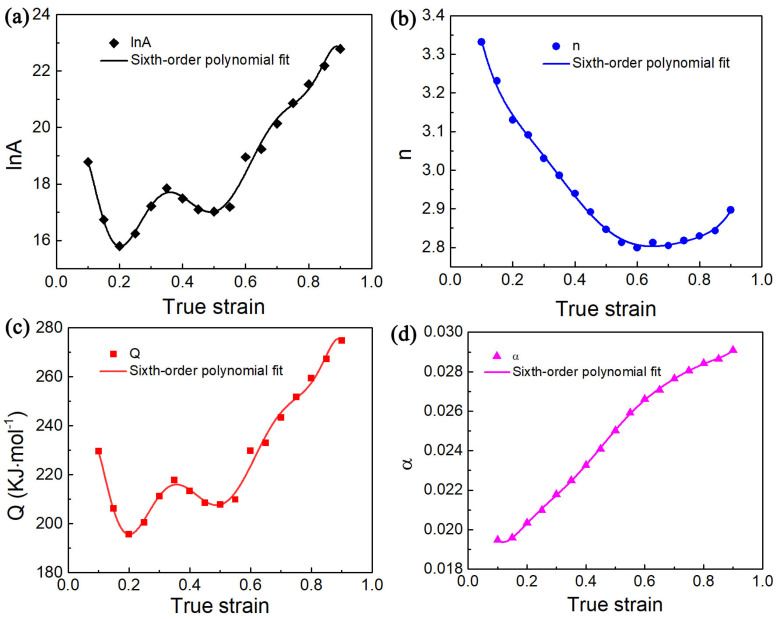
Correlation fitting curves between four material parameters ((**a**) ln *A*; (**b**) *n*; (**c**) *Q* and (**d**) *α*)) and strain value by employing the sixth-order polynomial fitting method in the single-phase region.

Nevertheless, Equation (1) fails to account for the effect of strain on flow stress under hot deformation. Recent researchers have found that deformation strain has a notable influence on the material parameters in the constitutive model [31,32]. The relationship between material parameters for different strains must be obtained to improve the reliability of flow stress estimation for Ti65 alloy, as shown in Figure 11 and Figure 12. The results exhibit substantial fluctuations in values of the four material parameters in relation to strain. The relationship between material parameters and strain can be calculated using Formula (11). Finally, Formula (12) can be used to calculate the flow stress value. The coefficients of polynomial function are displayed in Table 2 and Table 3, corresponding to various phase regions, respectively.

(11) $$\left[\begin{array}{c} \ln\;A\\ \alpha \\ \begin{array}{c} n\\ Q \end{array} \end{array}\right]=\left[\begin{array}{cc} \begin{array}{c} \begin{array}{c} B_{0}\\ C_{0} \end{array}\\ D_{0} \end{array} & \begin{array}{c} \begin{array}{c} \begin{array}{cc} \cdots & B_{6} \end{array}\\ \begin{array}{cc} \cdots & C_{6} \end{array} \end{array}\\ \begin{array}{cc} \cdots & D_{6} \end{array} \end{array}\\ E_{0} & \begin{array}{cc} \cdots & E_{6} \end{array} \end{array}\right]\left[\begin{array}{c} \varepsilon^{0}\\ \varepsilon^{1}\\ \begin{array}{c} \vdots \\ \varepsilon^{6} \end{array} \end{array}\right]$$

(12) $$\sigma =\frac{1}{\alpha }ln\left\{{\left(\frac{Z}{A}\right)}^{\frac{1}{n}}+{\left[{\left(\frac{Z}{A}\right)}^{\frac{2}{n}}+1\right]}^{\frac{1}{2}}\right\}$$

**Figure 13:**
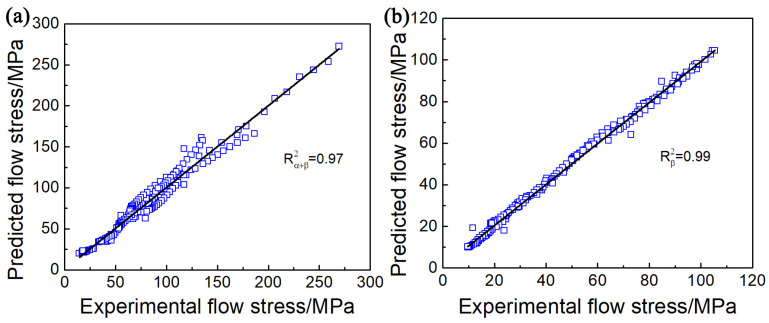
The relevance of the correlation between the experimental flow stress and the estimated flow stress for Ti65 alloys with different phase fields using an Arrhenius-type constitutive model: (**a**) α + β phase region, (**b**) single β phase region.

Figure 13a,b illustrates the measured and estimated flow stress values of the Ti65 alloy using the Arrhenius type constitutive model in different phase regions, respectively. In addition, the correlation coefficient (*R*) and the average absolute relative error (*AARE*) are used to demonstrate the precision of the established model:

(13) $$R=\frac{\sum_{i=1}^{N}\left(X_{i}-\bar{X})(Y_{i}-\bar{Y}\right)}{\sqrt{\sum_{i=1}^{N}{\left(X_{i}-\bar{X}\right)}^{2}\sum_{i=1}^{N}{\left(Y_{i}-\bar{Y}\right)}^{2}}},$$

(14) $$AARE=\frac{1}{N}\sum_{i=1}^{N}\left|\frac{Y_{i}-X_{i}}{X_{i}}\right|\times 100\%,$$

where $X_{i}$ is the measured flow stress value, and $\bar{X}$ is the average of the measured flow stress values; $Y_{i}$ is the estimated value of flow stress, and $\bar{Y}$ is the average of the values of estimated flow stress; *N* represents the entirety of the data set.

For the two-phase region, *R* and *AARE* are 0.97 and 11.01%. For the single-phase region, the two values above are 0.99 and 3.93%. It is worth mentioning that the constitutive equation that considers the effect of strain based on the Arrhenius type allows for a more accurate prediction of the flow stresses in the single-phase region for Ti65 alloys compared to the two-phase region.

### 3.3. Grey Wolf Optimization–Back-Propagation Neural Network Model

**Figure 14:**
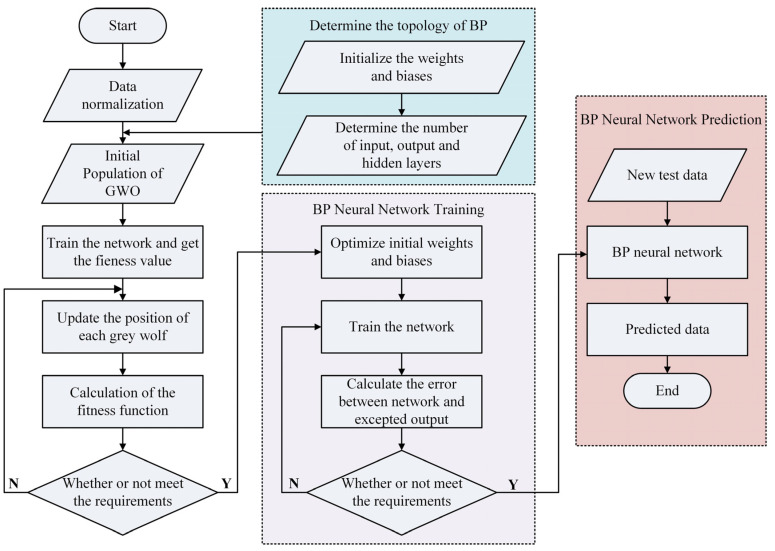
Flow chart of the proposed procedure for the GWO–BP machine learning algorithm.

The back-propagation neural network is a highly popular model in the field of machine learning [33]. Presently, the prevailing models, employed for estimating the flow characteristics of materials, typically consist of three layers. However, the prediction accuracy is greatly influenced by factors such as the initial weights, the threshold value, and the number of neural nodes in the hidden layers. The input layer comprises the variables, including strain, strain rate, and temperature, whereas the output layer represents the flow stress value. The artificial neural network utilizes numerous nonlinear relationships to connect the given input data with the outcome data. There is no necessity to create a model based on mathematics for connecting the input and output data. This study employed a Grey Wolf optimization–back-propagation neural network model (GWO–BP) comprising a layer for input, a layer for output, and one or more hidden layers. Generally speaking, the greater the number of hidden layers, the greater the amount of data computation, which will lead to the reduction of computing efficiency. In this paper, the GWO algorithm was used to optimize the single hidden layer neural network to achieve high efficiency. The number of nodes in the hidden layers can be calculated by Equation (15), as follows:

(15) $$N=\sqrt{n+m}+k$$

where *n* is the number of input layer nodes, and *m* is the output layer nodes. *k* is the integer ranging from 2 to 10 in this research, and the number of hidden layer neurons in this ranges from 4 to 12. Therefore, the GWO–BP model has great precision in predicting the flow stress during thermal compression deformation. Figure 14 illustrates the current selection procedure of the GWO–BP algorithm.

To ensure the precision and stability of the analysis, the input data must be normalized before training the network. This is because different factors in hot compression deformation have unique physical meanings, and normalizing the data helps reduce their influence on the results and improve convergence reliability. The subsequent equation was extensively employed for the normalization process:

(16) $$x_{i}=0.1+0.8\left(\frac{x_{i}^{\prime }-x_{min}}{x_{max}-x_{min}}\right),$$

where $x_{i}^{\prime }$ is the experimental value, and $x_{i}$ is the normalized data correlating with $x_{i}^{\prime }$. All the strain rates used in this experiment are 0.01–10 s^−1^ and exhibit significant variation, making them unsuitable for normalization using Equation (17). Thus, the logarithmic function approach is utilized for the strain rate.

(17) $$\dot{\varepsilon_{\dot{i}}}=0.1+0.8\left(\frac{lg\dot{\varepsilon_{\dot{i}}^{\prime }}-lg{\dot{\varepsilon }}_{min}}{lg{\dot{\varepsilon }}_{max}-lg{\dot{\varepsilon }}_{min}}\right),$$

where $\dot{\varepsilon_{\dot{i}}^{\prime }}$ is a value of the experimental strain rate, and $\dot{\varepsilon_{\dot{i}}}$ is a value of normalized data. The GWO–BP model was trained using a random selection of 75% from the experimental datasets. The remaining 25% of the datasets were used to assess the efficiency of the established model, specifically within a strain range of 0.05 to 0.9. Tansig and purlin are the activation function used in the hidden and output layer, respectively. Trainlm is employed as a training function. The GWO–BP algorithm has the following settings: the model’s maximum number of repetitions is set to 2000, with a target error of 10^−6^, and the learning rate is set at 0.01. Root mean square error (*R_MSE_*) calculated by Equation (18) was used to determine the appropriate number of hidden layer nodes of GWO–BP.

(18) $$R_{MSE}=\sqrt{\frac{1}{N}}\left|\sum_{i=1}^{N}\left(E_{i}-P_{i}\right)\right|$$

**Figure 15:**
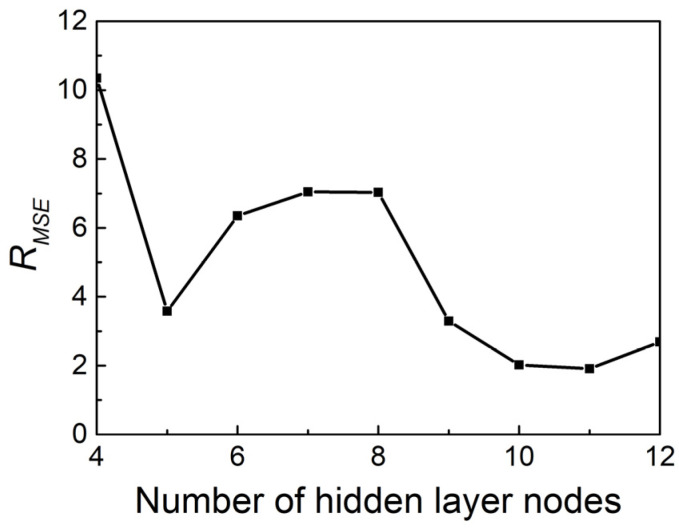
The relationship between *R_MSE_* and the number of hidden layer nodes.

**Figure 16:**
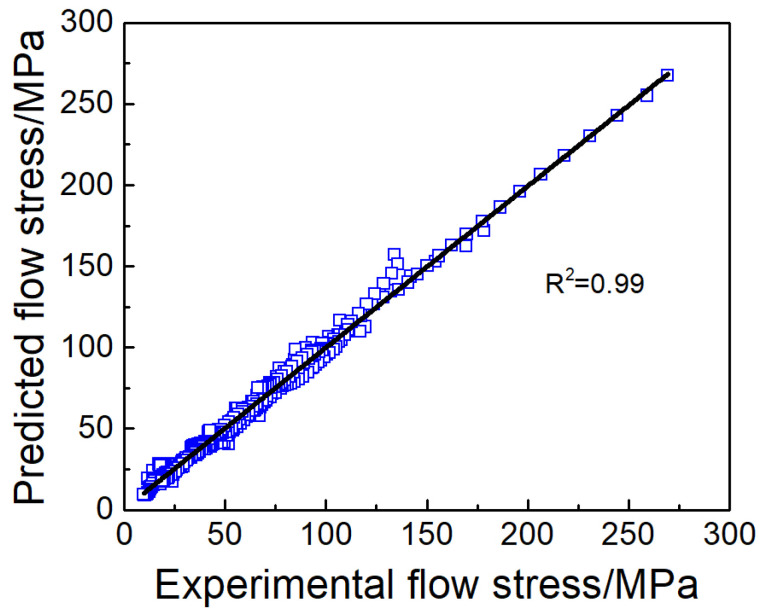
The relevance of the correlation between the actual experimental and estimated flow stress for Ti65 alloy under different deformation parameters analyzed using the GWO–BP machine learning algorithm.

The relationship between *R_MSE_* and the number of hidden layer nodes is displayed in Figure 15, and it is obvious that, when the number of neuron nodes in the hidden layer is 11, *R_MSE_* reaches the minimum value, indicating that the prediction accuracy of the model is highest currently. Figure 16 depicts the relationship between the measured and estimated Ti65 alloy flow stress utilizing the GWO–BP machine learning algorithm. The *R* and *AARE* using this method are 0.994 and 1.55%, respectively. Upon comparing the actual flow stress with the estimated flow stress, obtained from the Arrhenius type and the GWO–BP machine learning model, the GWO–BP model provides superior results in the α + β phase region compared to the Arrhenius constitutive equation. The aforementioned results show that the GWO–BP (ANN) model is capable of accurately predicting the values of heat distortion flow stress in Ti65 alloy.

### 3.4. Microstructure Evolution

**Figure 17:**
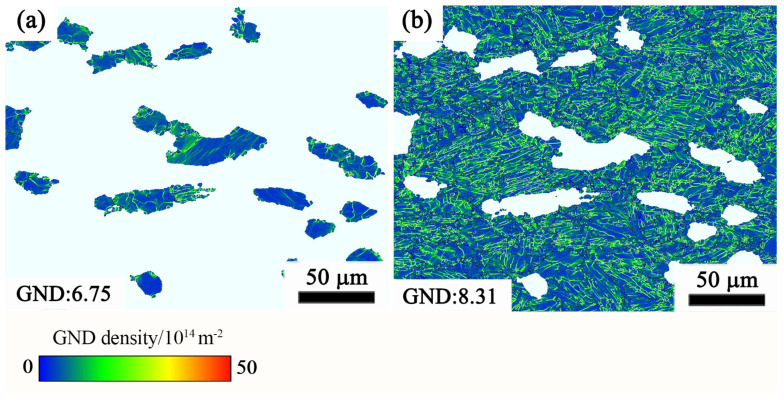
GND density maps of α_p_ and α_s_ phases with true strain of 0.35 under the strain rate of 0.01 s^−1^ and the temperature of 950 °C: (**a**) α_p_ phase; (**b**) α_s_ phase.

From the aforementioned flow stress–strain curves, it is evident that, as deformation continues beyond the peak stress value, progressive softening processes emerge, leading to a change in the flow behavior. The softening mechanisms of near-α Ti alloys can be attributed to a variety of factors including DRX, DRV, dynamic spheroidization, super-plasticity, flow localization, deformation bands, and cracking [34,35,36,37]. The stress–strain data offer a depiction of the correlation between the stress value and thermal characteristics. However, accurately determining the deformation mechanism from stress–strain data alone is a difficult task. Therefore, a thorough examination will be carried out to investigate the mechanism by which the flow softens, together with the changes in the microstructure that occur under various deformation situations. Dislocation density analysis in the early stage of α_p_ and α_s_ phase when the deformation amount is 30% (the corresponding true strain is 0.35) is shown in Figure 17. Compared to the matrix β phase, in general equiaxed α particles act as hard inclusions and β phase is easier to deform than equiaxed α, which has been demonstrated by researchers [25,38]. The orientation relationship between secondary alpha phase (α_s_) and β phase follows the Burgers orientation relationship (BOR), i.e., {0001}α||{110}β and <11–20>α||<111>β, and the slip system between them maintains a parallel relationship, which can make the slip easily transfer across the interface [39]. Therefore, there are more substructures in the secondary α phase than in the primary α phase, as can be seen in Figure 17a,b.

#### 3.4.1. Influence of Temperatures during Deformation on the Microstructure

**Figure 18:**
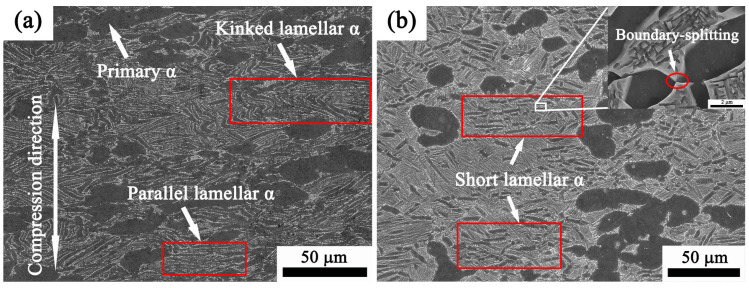
Microstructure of Ti65 alloy subjected to compression at 0.1 s^−1^ in two-phase region under different temperatures: (**a**) 950 °C, (**b**) 980 °C.

Figure 18 shows the effect of temperatures during deformation on the microstructure of Ti65 at a strain rate of 0.1 s^−1^. The deformation temperature has a major impact on the evolution of the microstructure. The shape and spatial arrangement of the lamellar α have changed considerably compared to the initial bimodal microstructure (Figure 1). As shown by the red circle in Figure 18a, the lamella α exhibits a concentrated distribution pattern vertical to the compression direction during deformation at a temperature of 950 °C. The transition from a uniform distribution (original microstructure) to a centralized distribution suggests that the lamellar α-structure will gradually rearrange itself perpendicular to the compression axis during deformation [40]. Comparing Figure 18a,b, it can be found that, when the deformation temperature rises, the volume proportion of the α_p_ gradually decreases, and the grain morphology also changes gradually, becoming increasingly equiaxed with a smaller aspect ratio. This may be due to the microstructure coarsening of primary α induced by element diffusion during hot deformation [41]. In addition, certain structures exhibit bending or kinking, as highlighted by the red circle in Figure 18a. The lamellar α phase exhibits significant heterogeneity in terms of its spatial trace orientation, bending, and kinking. The grain shape of the α colony during the compression depends mainly on the angle between the c-axis of the α phase and the compression axis. Thomas et al. [42] demonstrated that, when the c-axis of α phase in the colonies is parallel to the loading direction, the lamellar α phase displays a hard orientation, which is prone to distortion or bending during the subsequent thermal deformation and is not susceptible to spheroidization. As the temperature increased to 980 °C, seen in Figure 18b, the grain of the partial lamellar became short-bar in morphology, and the aspect ratio decreased. Formation of a short bar-like α phase may be a prerequisite for the occurrence of spheroidization, which can be seen as localized enlargement in the top right corner of Figure 18b, and boundary-splitting can be demonstrated in the present study for dynamic globularization. The process of boundary-splitting frequently takes place during the hot deformation and heat treatment of near-α and α + β phase titanium alloys [11,43]. Dislocation interfaces in lamellar α are first formed during the deformation, and the appearance of the sub-boundaries disrupts the surface tension equilibrium at the borders between the α and β phases. As a result, the sub-boundary becomes thermodynamically unstable, and a heat cup is formed. Then, α/α sub-boundaries are penetrated by β phase through the site of thermal cups and separate the lamellar α into bamboolike morphology or a near-equiaxed shape. Finally, with the further migration of β-phase stabilizing elements, dynamic globularization of lamellar α can develop.

**Figure 19:**
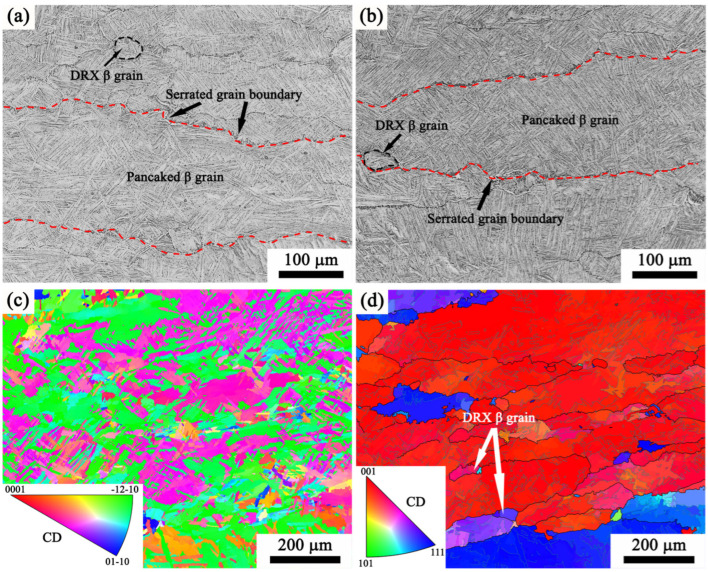
Microstructure of Ti65 alloy subjected to compression at 0.1 s^−1^ in the single-phase region: (**a**) 1050 °C, (**b**) 1110 °C, (**c**) the inverse pole figure map of α_S_ precipitated in β grains under deformation at 1110 °C, (**d**) reconstruction IPF map of high temperature β grains corresponding to (**c**).

Figure 19 illustrates the microstructure evolution after deformation in the single-phase region under different temperatures using analysis of optical image (OM) and electron backscatter diffraction (EBSD) studies. The microstructure of the central region of the specimen distinctly showed that flattened primary β grains were vertical to the compression axis. Additionally, the length dimension of the grain size of β phase increases in a scale of millimeters. The grain boundaries display a serrated morphology, with only a few recrystallized grains observed in the localized regions of the serrated grain boundaries. As stated in Section 3.2 above, *Q* in the single-phase field is 238.728 kJ/mol, similar to the reported range of 152–250 kJ/mol for pure titanium’s self-diffusion [26,44]. At low strain rates, the main mechanisms of deformation in the single β-phase field are dynamic recovery and strain-induced grain boundary migration. These mechanisms have been observed in other near-α titanium alloys, such as Ti60 [34] and IMI834 [45].

#### 3.4.2. Influence of Strain Rates during Deformation on the Microstructure

**Figure 20:**
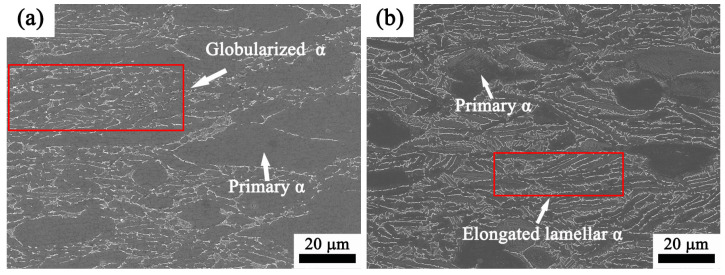
Microstructure of Ti65 alloy compressed at 950 °C in the two-phase region under various strain rates: (**a**) 0.01 s^−1^, (**b**) 1 s^−1^.

The microstructures of the Ti65 alloy deformed at various strain rates at a temperature of 950 °C are depicted in Figure 20a,b. As shown in Figure 18a, it is also clear from Figure 20a,b that the strain rate has no substantial effect on the thickness of the lamellar α phase. The aspect ratio of the primary α phase dropped as the strain rates increased due to the diffusion caused by the generated deformation heat. During the heated deformation at a greater strain rate, the heat created is unable to dissipate because of the combination of low thermal conductivity and the limited time available for the heat transfer [46]. The proportion of globularized lamellae α increased as the strain rate dropped at a deformation temperature of 950 °C, as depicted in Figure 20a,b. This observation is linked to the mechanism, which involves the splitting of boundaries and migration of terminations [47].

**Figure 21:**
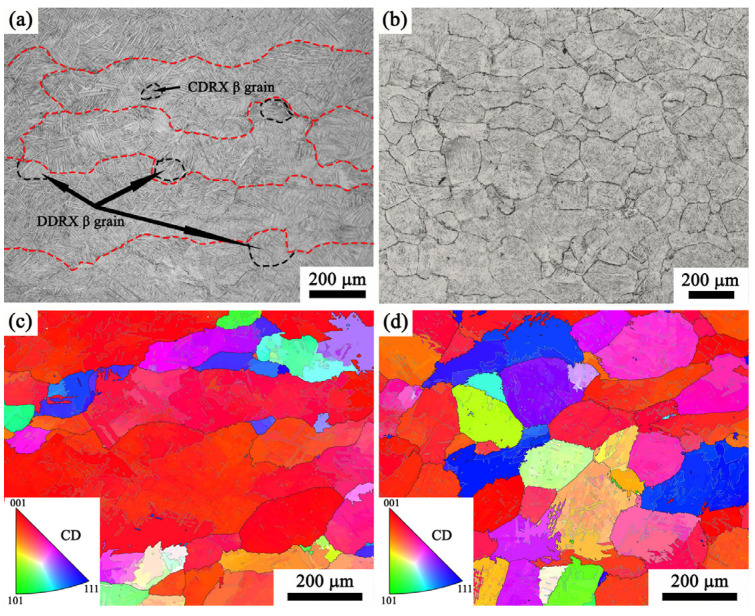
Microstructure of Ti65 alloy compressed at 1110 °C in the single β phase: (**a**) 0.01 s^−1^, (**b**) 10 s^−1^, (**c**) reconstruction IPF map of high temperature β grains under the strain rate of 0.01 s^−1^; (**d**) reconstruction IPF map of high temperature β grains under the strain rate of 10 s^−1^.

Figure 21 shows that there is an observable dynamic recrystallization (DRX) process occurring as the strain rate increases in the single-phase of the Ti65 alloy. In the CDRX process, sub-grains are primarily formed through the dislocation rearrangement at low-angle grain boundaries (LAGBs). Eventually, new dynamic grains containing high-angle grain boundaries (HAGBs) are generated through the gradual rotation of these sub-grains [48]. The DDRX process exhibits a clearly identifiable nucleation step characterized by the bulging and movement of grain borders, which is then followed by the formation of additional grains [49]. Upon comparing Figure 21a,b, it is evident that the proportion of dynamic recrystallization rises as the deformation rate increases. The restriction of dynamic recrystallization at a lower strain rate can be ascribed to the challenge of retaining a sufficiently high dislocation density. Conversely, the dislocation density experiences a substantial rise at high strain rates. With the increase of strain rate, the shape of β grain gradually changes from flat to an equiaxed pattern. The CDRX mechanism typically results in a similar grain orientation between the newly formed grains and the initial β grains, resulting in notable anisotropy in the grain orientation spread. The DDRX mechanism induces a random crystal orientation in the newly recrystallized grains, hence reducing the anisotropy of the alloy [50]. Through the analysis of the IPF map of the reconstructed high-temperature β grains, the controlling mechanism of dynamic recrystallization during the high strain rates such as 10 s^−1^ may be DDRX.

## 4. Conclusions

A comprehensive investigation was conducted to examine the thermal deformation behavior and mechanism of a novel near-α titanium Ti65 alloy under various conditions, and the microstructure evolution during the compression was also studied based on different characterization methods. Additionally, constitutive models were developed using Arrhenius-type equations and machine-learning algorithms. The primary deductions can be depicted.

(1) The stress values of the Ti65 exhibit a reduction as temperatures increase and strain rates decrease. The softening trend of flow stress is more obvious in the two-phase region.
(2) Based on the experimental and predicted values of flow stress, the Arrhenius constitutive model considering strain compensation and the GWO–BP machine-learning model are compared and analyzed. The *R* of the Arrhenius constitutive equations was 0.974 in the two-phase region and 0.995 in the single-phase region, and the *R* of the GWO–BP machine-learning model is 0.994. For the deformation behavior of a single-phase region, both models have good prediction ability. However, for the two-phase deformation process, the prediction accuracy of GWO–BP is higher than that of the Arrhenius model.
(3) In the process of deformation in the two-phase region, the lamellar α phase changes from a twisted, long shape to a globular one along with strain rate decrease. Boundary-splitting, as the main spheroidization mechanism, is observed with increasing temperature.
(4) Dynamic recovery and strain-induced grain boundary migration are the main deformation mechanisms at low strain rates in the single-phase region. The degree of recrystallization increases obviously with the increase of strain rate, and the DDRX may be the dominant mechanism governing dynamic recrystallization at high strain rates.

## Figures and Tables

**Table 1:** Chemical composition of the Ti65 alloy (wt. %).

Al	Sn	Zr	Ta	W	Si	Nb	Mo	C	Ti
5.92	3.94	3.45	0.86	0.66	0.48	0.32	0.31	0.05	Bal.

**Table 2:** The material variables for Ti65 alloy determined using the polynomial fitting approach in the two-phase region.

Material Parameters	Sixth-Order Polynomial Fitting
*ln A*	$\ln A=165.68\;-\;698.39\varepsilon \;+2720.73\varepsilon^{2}\;-\;5551.85\varepsilon^{3}+6890.46\varepsilon^{4}\;-\;5111.94\varepsilon^{5}+1698.47\varepsilon^{6}$
*n*	$n\;=4.49\;-\;4.04\varepsilon \;+12.69\varepsilon^{2}\;-\;21.32\varepsilon^{3\;}+48.14\varepsilon^{4}\;-\;67.82\varepsilon^{5\;}+34.28\varepsilon^{6}$
*Q*	$Q\;=1763.35\;-\;7533.99\varepsilon \;+29,789.53\varepsilon^{2}\;-62,228.76\varepsilon^{3}+78,873.97\varepsilon^{4}\;-\;58,989.22\varepsilon^{5}+19,526.48\varepsilon^{6}$
*α*	$\alpha \;=0.01\;-\;0.05\varepsilon \;+0.37\varepsilon^{2}\;-\;1.19\varepsilon^{3}+2.01\varepsilon^{4}\;-\;1.66\varepsilon^{5}+0.54\varepsilon^{6}$

**Table 3:** The material variables for Ti65 alloy determined using the polynomial fitting approach in the single-phase region.

Material Parameters	Sixth-Order Polynomial Fitting
*ln A*	$\ln A=43.41\;-\;462.89\varepsilon \;+2954.12\varepsilon^{2}\;-\;9079.11\varepsilon^{3}+14,447.36\varepsilon^{4}\;-\;11,410.09\varepsilon^{5}+3538.48\varepsilon^{6}$
*n*	$n\;=3.94\;-\;10.16\varepsilon \;+55.59\varepsilon^{2}\;-\;170.80\varepsilon^{3}+276.45\varepsilon^{4}\;-\;221.96\varepsilon^{5}+70.13\varepsilon^{6}$
*Q*	$Q\;=509.29\;-\;5265.17\varepsilon \;+33,693.25\varepsilon^{2}\;-\;103,934.11\varepsilon^{3}+165,949.13\varepsilon^{4}\;-\;131,457.42\varepsilon^{5}+40,881.69\varepsilon^{6}$
*α*	$\alpha \;=0.02\;-\;0.03\varepsilon \;+0.25\varepsilon^{2}\;-\;0.72\varepsilon^{3}+1.22\varepsilon^{4}\;-\;1.09\varepsilon^{5}+0.39\varepsilon^{6}$

## Data Availability

The original contributions presented in the study are included in the article, further inquiries can be directed to the corresponding author.
